# Supplementary material for: Association between oral microbiome and five types of respiratory infections: a two-sample Mendelian randomization study in east Asian population
Source: Front Microbiol. 2024 Apr 10;15:1392473. doi: 10.3389/fmicb.2024.1392473 (PMC11039966; doi:10.3389/fmicb.2024.1392473)
Supplement: Supplementary file 1 [file Data_Sheet_1.zip › Supplementary Materials/MR plots of saliva/Pneumonia/s__Streptococcus_pneumoniae_D_mgs_1023/forest.pdf]

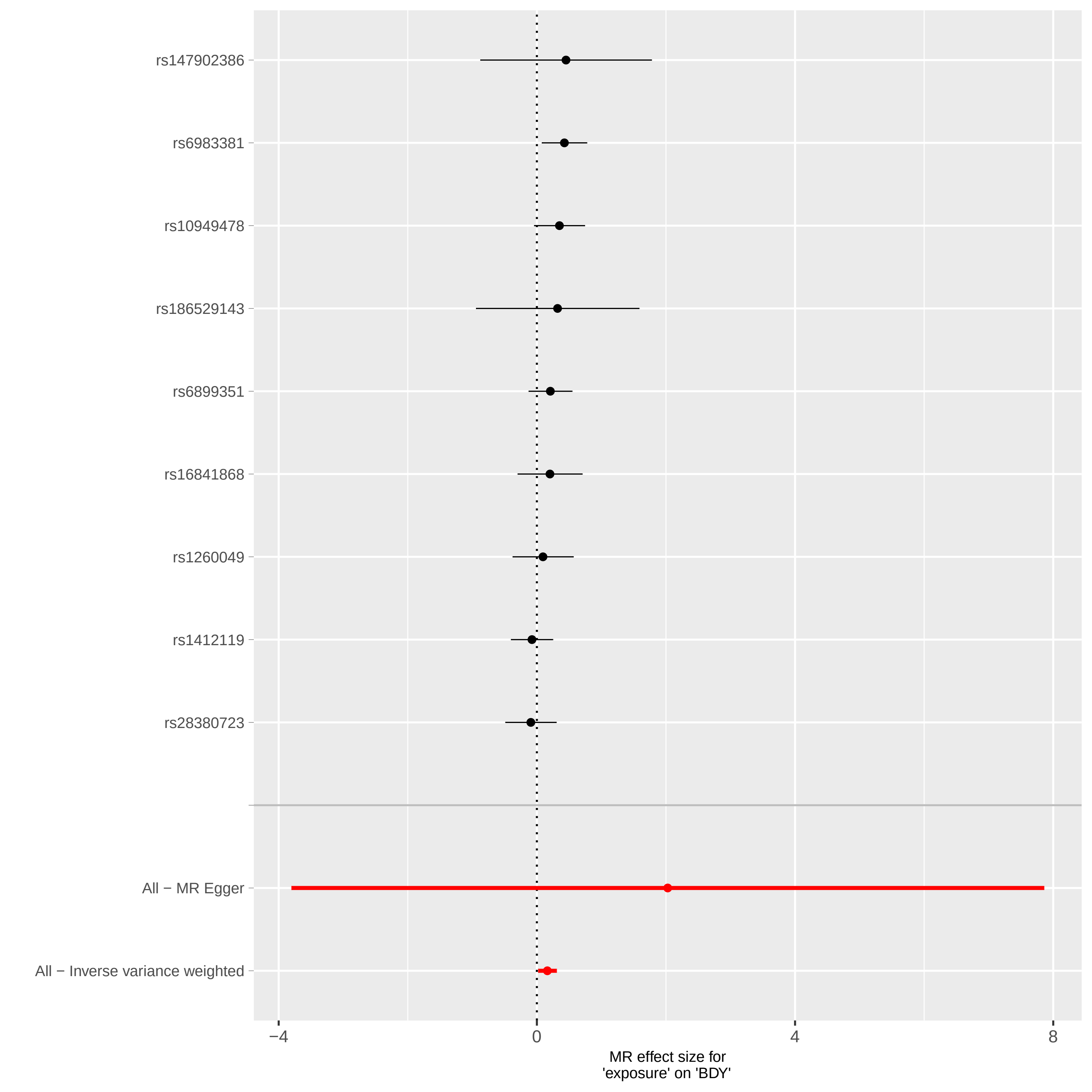

rs147902386
rs6983381
rs10949478
rs186529143
rs6899351
rs16841868
rs1260049
rs1412119
rs28380723
All − MR Egger
All − Inverse variance weighted
−4
0
4
8
MR effect size for
'exposure' on 'BDY'
